# Ecological characterization of 175 low‐pathogenicity avian influenza viruses isolated from wild birds in Mongolia, 2009–2013 and 2016–2018

**DOI:** 10.1002/vms3.1281

**Published:** 2023-09-28

**Authors:** Ariunbaatar Barkhasbaatar, Martin Gilbert, Amanda E. Fine, Enkhtuvshin Shiilegdamba, Batchuluun Damdinjav, Bayarbaatar Buuveibaatar, Bodisaikhan Khishgee, Christine K. Johnson, Connie Y. H. Leung, Ulaankhuu Ankhanbaatar, Dulam Purevtseren, James M. Tuttle, Jonna A. K. Mazet, Joseph S. Malik Peiris, Losolmaa Jambal, Munkhduuren Shatar, Tuvshintugs Sukhbaatar, Sarah H. Olson

**Affiliations:** ^1^ Wildlife Conservation Society Mongolia Program Ulaanbaatar Mongolia; ^2^ Cornell Wildlife Health Center College of Veterinary Medicine Cornell University, New York Ithaca USA; ^3^ Wildlife Conservation Society Health Program Bronx New York USA; ^4^ Division of Transboundary Animal Viral Diseases Diagnosis and Surveillance State Central Veterinary Laboratory Ulaanbaatar Mongolia; ^5^ General Authority for Veterinary Services Ulaanbaatar Mongolia; ^6^ EpiCenter for Disease Dynamics, One Health Institute, School of Veterinary Medicine University of California Davis California USA; ^7^ Centre for Comparative Medicine Research, Li Ka Shing Faculty of Medicine The University of Hong Kong Hong Kong People's Republic of China; ^8^ Southern Arizona Veterinary Specialty & Emergency Center Tucson Arizona USA; ^9^ Karen C. Drayer Wildlife Health Center, One Health Institute University of California Davis California USA; ^10^ School of Public Health, Li Ka Shing Faculty of Medicine The University of Hong Kong Hong Kong People's Republic of China; ^11^ Wildlife Science and Conservation Center of Mongolia Ulaanbaatar Mongolia

**Keywords:** Avian influenza, characterization, low pathogenicity, Mongolia, wild birds

## Abstract

**Background:**

Since 2005, highly pathogenic avian influenza A H5N1 viruses have spread from Asia worldwide, infecting poultry, humans and wild birds. Subsequently, global interest in avian influenza (AI) surveillance increased.

**Objectives:**

Mongolia presents an opportunity to study viruses in wild birds because the country has very low densities of domestic poultry and supports large concentrations of migratory water birds.

**Methods:**

We conducted AI surveillance in Mongolia over two time periods, 2009–2013 and 2016–2018, utilizing environmental fecal sampling. Fresh fecal samples were collected from water bird congregation sites. Hemagglutinin (HA) and neuraminidase (NA) subtypes of positive samples were identified through viral isolation or molecular assays, with pathogenicity determined by HA subtype or sequencing the HA cleavage site.

**Results:**

A total of 10,222 samples were collected. Of these, 7,025 fecal samples were collected from 2009 to 2013, and 3,197 fecal samples were collected from 2016 to 2018. Testing revealed 175 (1.7%) positive samples for low‐pathogenicity influenza A, including 118 samples from 2009 to 2013 (1.7%) and 57 samples from 2016 to 2018 (1.8%). HA and NA subtyping of all positives identified 11 subtypes of HA and nine subtypes of NA in 29 different combinations. Within periods, viruses were detected more frequently during the fall season than in the early summer.

**Conclusion:**

Mongolia's critical wild bird habitat is positioned as a crossroad of multiple migratory flyways. Our work demonstrates the feasibility of using an affordable environmental fecal sampling approach for AI surveillance and contributes to understanding the prevalence and ecology of low‐pathogenicity avian influenza viruses in this important location, where birds from multiple flyways mix.

## Graphical abstract

- Collected a total of 10,222 fecal samples from wild birds in Mongolia between 2009–2013 and 2016–2018 and found 175 low‐pathogenicity avian influenza viruses of 29 subtype combinations.
- During these time periods, the viruses were more frequently detected in the fall season (August to October) compared with the early summer (April to July).
- The study demonstrates the use of a cost‐effective fecal sampling approach for monitoring avian influenza in wild bird populations and contributes to our understanding of the prevalence and ecology of these viruses in Mongolia, a country with a globally important habitat for large concentrations of migratory water birds.

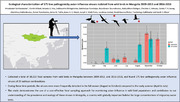

## 1 INTRODUCTION

Influenza A viruses are pathogens of global concern in both human and veterinary medicine (Olson et al., 2014; Webster et al., 1992; Wiethoelter et al., 2015). Avian influenza virus (AIV) subtypes are generally classified into two groups based on their pathogenicity in domestic poultry: high‐pathogenicity avian influenza viruses (HPAIVs) and low‐pathogenicity avian influenza viruses (LPAIVs). Subtypes are characterized based on the antigenic profile of their surface proteins hemagglutinin (HA, 16 subtypes, H1–H16) and neuraminidase (NA, nine subtypes, N1–N9) (Foster, 2018; Fouchier et al., 2005; Krauss et al., 2004; Swayne, 2007). Highly pathogenic strains can cause high rates of mortality in domestic poultry flocks and can lead to large outbreaks with severe economic consequences in affected countries (Sonnberg et al., 2013). In the spring of 1996, domestic geese in Guangdong, China, were affected by HPAI associated with haemorrhagic and neurological disease. The causal agent was identified as an HPAIV strain designated as A/goose/Guangdong/1996‐like (H5N1) (Wan, 2012). In late 2002 and early 2003, A/goose/Guangdong/1996‐like (H5N1) HPAI viruses were associated with the mortality of wild and captive water birds in Hong Kong parklands, representing the first reported detections of HPAI in wild birds since 1961 (Ellis et al., 2004; Rowan, 1962). In 2005, HPAIs were associated with an outbreak among migratory birds on Lake Qinghai, China, in May and June, during which more than 6,000 birds died (J. et al., 2005). Since 2005, surveillance of wild birds for AIV has increased worldwide, tracking the spread of the A/goose/Guangdong/1996‐like (H5N1) HPAIVs among domestic and wild birds in Asia, Europe, Africa and North America (Machalaba et al., 2015).

**FIGURE 1:**
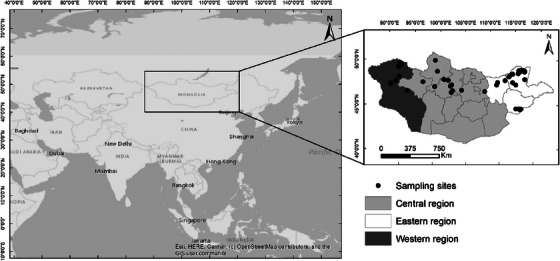
Locations of sampling sites for avian influenza surveillance in Mongolia, 2009–2018.

Within Asia, Mongolia is a particularly ideal location to study the dynamics of wild bird influenza viruses, in part because the country has very low densities of domestic poultry (*FAO Statistics*, n.d.). Mongolia also supports large populations of wild birds from two major migratory flyways and has important breeding, moulting and pre‐migratory staging areas (Batbayar & Natsagdorj, 2009; Newton, 2008). The relative absence of poultry within the country provides a near unique opportunity to study the epidemiology of the virus in the absence of domestic influence (Gilbert et al., 2012; Spackman et al., 2009). The migration of wild birds contributes to the global movement of these viruses (Reed et al., 2003). AIV surveillance enhances our understanding of the spread of influenza viruses around the world, from documenting outbreaks in domestic poultry or transmission from poultry to humans to identifying potential exposure of wild birds to highly pathogenic influenza viruses from poultry. In addition, AIV surveillance in wild birds aids our understanding of the dynamics and viral ecology in the natural hosts, capturing the viral diversity in these reservoirs, which can improve our understanding of influenza A virus risk in other species (Diskin et al., 2020). We report the detection of 175 LPAIVs isolated from wild birds in Mongolia. The surveillance was conducted throughout Mongolia in two time periods representing 8 years in the period 2009–2018 (Figure 1).

## 2 MATERIALS AND METHODS

### 2.1 Study area and period

Lying between latitudes 42°N and 51°N, Mongolia is a vast land‐locked country extending east–west across 2,500 km of Central Asia between Russia and China, with an approximate area of 1.5 million km^2^. Although a country of climatic and geographic extremes (Wingard J.R. and P. Zahler, 2006), much of the land area consists of open steppe, transitioning to taiga forest in the north and the Gobi Desert in the south and west. Wetlands and lakes of variable size fleck the landscape, with approximately 3,000 rivers stretching over 67,000 km in the north (Wingard J.R. and P. Zahler, 2006). Sampling sites were generally selected based on known water bird congregation points. During 2009–2013, samples were collected annually at 34 locations in total throughout the country in spring, summer and autumn. However, during 2016–2018, samples were collected at 25 locations in total across Mongolia during the spring and fall seasons each year (Figure 1).

### 2.2 Fecal sample collection

Fecal samples were collected from three ecogeographic regions: steppe (eastern region), mountain steppe (central region) and desert steppe (western region). Before fresh fecal sample collection, we observed and identified bird species throughout the water body and then selected potential sampling areas. Fresh fecal samples were collected where water birds were observed congregating or roosting. Fecal sampling focused on single‐species or single‐genus flocks to enable the identification of the species being sampled. Between 2009 and 2013, fecal sampling was focused on the order Anseriformes (particularly dabbling ducks and shelducks), and the host species identity of all AIV‐positive samples was confirmed using DNA barcoding (Cheung et al., 2009). Sampling from 2016 to 2018 was targeted on orders Anseriformes and Charadriiformes, and species identification was based on field observation. In addition, we collected small numbers of samples from order Gruiformes (*n* = 10, cranes) in 2016–2018. Samples were stored individually in 2.0 ml cryovials containing viral transport media (VTM) between 2009 and 2013, and between 2016 and 2018, samples were stored in 2.0 ml VTM and 2.0 ml TRIzol (Leung et al., 2007). Samples were initially maintained at 4°C and then frozen in liquid nitrogen within four hours of collection. Care was taken to maintain the cold chain for specimens throughout transport, and once in the lab, all samples were stored at −70°C or below until processed.

### 2.3 Laboratory protocols for 2009–2013 surveillance

Specimens were inoculated into 9‐ to 10‐day‐old embryonated specific‐pathogen‐free eggs and incubated at 37°C for three days. The allantoic fluid harvested from egg cultures was then tested by the hemagglutinin assay (Swayne, 1998). Positive samples in HA were extracted with viral RNA extraction kits (Qiagen, Hilden, Germany) according to the manufacturer's instructions. One‐step RT‐PCR was performed using SuperScript® III One‐Step RT‐PCR systems with Platinum® Taq polymerase (Invitrogen) using primers designed for the conserved sequence for the HA2 coding region (Phipps et al., 2004) and NA gene (Fereidouni et al., 2009). Conventional RT‐PCR was performed in GeneAmp PCR systems 9700 (Applied Biosystems). Correct size bands were excised and purified using a gel purification kit and a DNA purification kit (Qiagen, Hilden, Germany). Sequencing was performed using DNA Analyser 3730xl (Applied Biosystems), and the HA and NA subtypes were confirmed and identified using the NCBI Blast tool. The pathogenicity of the isolates was determined by studying the sequence at the HA cleavage site.

### 2.4 Laboratory protocols for 2016–2018 surveillance

At the State Central Veterinary Laboratory in Mongolia, RNA was extracted from the TRIzol sample using the RNA MiniPrep Kit (Sigma‐Aldrich), and cDNA was transcribed using the SuperScript III First‐Strand cDNA Synthesis System (Invitrogen). Avian influenza RNA was detected with two assays. All samples collected from 2016 to 2018 were screened for the M gene (Anthony et al., 2012) with a universal control, and samples collected in 2017–2018 were additionally screened for the Orthomyxoviridae PB1 gene with a specific influenza A control (Lee et al., 2020). Each test batch included distilled water as a negative control. PCR products were visualized using 1.5% agarose gels. Correct size bands were excised, cloned and sequenced by Sanger dideoxy sequencing. Samples with genetic sequences confirmed as influenza A using the NCBI Blast tool were then subtyped by reverse transcription‐PCR (RT‐PCR). An assay targeting the HA1 coding region was used to identify H1–H16 hemagglutinin subtypes (Lee et al., 2001), and an assay targeting the NA gene was used to identify N1–N9 neuraminidase subtypes with distilled water as a negative control (Qiu et al., 2009). The RT‐PCR product was visualized on a 2% agarose gel with a 100 bp DNA ladder (Thermo Fisher Scientific), and the length of the product was used to identify a specific HA or NA subtype. Low pathogenicity was determined based on the absence of H5 or H7 subtypes.

## 3 RESULTS

A total of 10,222 fecal samples were collected from wild birds in Mongolia and tested for AIV. Of these, 7,025 samples were collected from 2009 to 2013, and 3,197 samples were collected from 2016 to 2018. A total of 175 samples (1.7 %) were positive for low‐pathogenicity influenza A viruses, and the annual proportion was between 0.5% and 3.9% (Table 1). Eleven subtypes of HA (H1–H8; H10–H12) and nine subtypes of NA (N1–N9) were identified in 29 different combinations. The most common subtypes of HA were H3 and H4, followed by H2, H10 and H1. For the NA subtypes, N8 and N6 were the most common, followed by N1, N2, N5 and N7. Subtyped cases were most often found in Anseriformes (*n* = 28), and the lowest number of subtypes were identified from Charadriiformes (*n* = 3). The most frequently detected subtype combinations in wild birds were H3N8 (*n* = 69; 39.4%), H4N6 (*n* = 31; 17.7%) and H10N7 (*n* = 9; 5.1%). The H4N6 subtype was detected in all years, and H3N8 in all but one of the years (Table 1). In contrast, the majority of subtypes were found in only a single year (*n* = 19). The highest number of subtypes were identified in the eastern region with 27 subtypes detected compared with nine in the central and four in the western regions (Supporting Information S1). No AIVs were detected in association with a wild bird mortality event.

**TABLE 1:** Number of viruses and subtype combinations detected in wild birds in Mongolia, 2009–2018.

	No. of samples		1,001	1,612	2,400	810	1,202	1,170	1,062	965
	Proportion of positives		2.0%	1.4%	1.1%	2.0%	2.8%	3.9%	0.6%	0.5%
	Subtype	Total detection years	2009	2010	2011	2012	2013	2016	2017	2018
Anseriformes	H1N1	3		5		1	2			
	H2N2	2					1		1	
	H2N3	1		1						
	H2N5	1					6			
	H3N1	2	1	3						
	H3N2	3			2	1	2			
	H3N3	1			1					
	H3N5	1					3			
	H3N6	2	1				1			
	H3N8	7	9	1	13	6	9	27		4
	H3N9	1				1				
	H4N3	1	1							
	H4N6	8	4	3	7	2	2	8	4	1
	H5N1	1					1			
	H5N3	1			2					
	H6N2	2				1		3		
	H6N6	1			1					
	H7N1	1		4						
	H7N3	1	1							
	H7N7	1		1						
	H8N4	1		1						
	H8N6	1		1						
	H10N7	2		1				8		
	H10N8	2	3	2						
	H11N2	1				1				
	H11N9	1				1				
	H12N1	1					2			
	H12N5	1					1			
	Subtotal		20	23	26	14	30	46	5	5
Charadriiformes	H2N2	1							1	
	H2N5	1				1				
	H2N6	1					3			
	Subtotal		0	0	0	1	3	0	1	0
Unknown	H3N1	1				1				
	Subtotal		0	0	0	1	0	0	0	0
	Total viruses		20	23	26	16	33	46	6	5
	No. of subtypes		7	11	6	10	12	4	3	2

**FIGURE 2:**
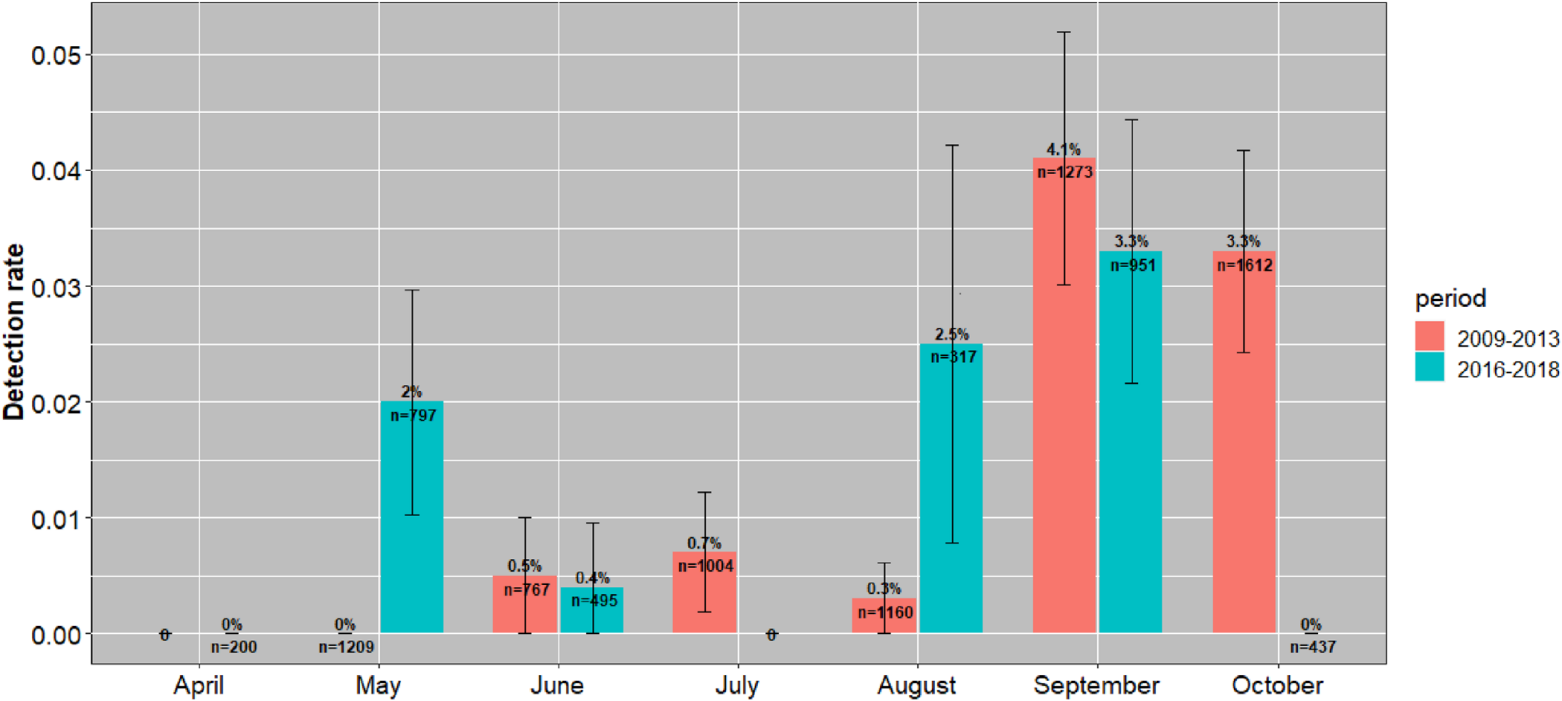
Detection prevalence of AIVs with standard deviations in wild birds by month in Mongolia from 2009 to 2018.

**FIGURE 3:**
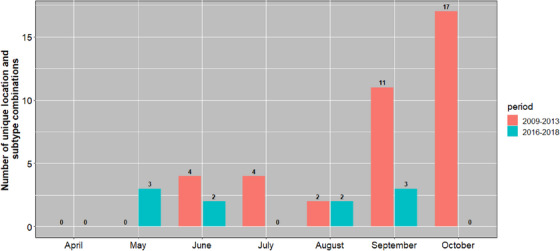
Number of unique location and subtype events detected in wild birds in Mongolia from 2009 to 2018.

Fecal samples were collected throughout eastern (67.8%), central (22.2%) and western (16%) regions of Mongolia. AIVs were detected relatively more frequently in the central (3.2%; 95% CI 2.5%–3.9%; 72/2,264) region compared with the eastern (1.5%; 95% CI 1.2%–1.8%; 93/6,319) and the western (0.6%; 95% CI 0.2%–1.1%; 10/1,639) regions (Figure 1). The highest proportion of influenza virus detections between 2009 and 2013 was found in September (4.1%) and October (3.3%), and the lowest in May (0.0%). In contrast, in 2016–2018, the highest proportion was in September (3.3%) and August (2.5%), and the lowest in April and October (0.0%). However, across both surveillance periods, the fall season, including the months of August, September and October, tended to have more positives (2.5%; 95% CI 2.1%–2.9%; 146/5,750) than the early summer (0.6%; 95% CI 0.4%–0.9%; 29/4,472), which included the months of April, May, June and July (Figure 2). Over the two periods, the occurrence of unique location and subtype combinations, as an indicator of local outbreaks, varied between 0 and 17. The 2009–2013 period showed local outbreaks peaked in September and October, whereas in the 2016–2018 period, local outbreaks were relatively stable through the months, with a striking absence in October (Figure 3).

Among the positives, Anseriformes [tufted duck (*Aythya fuligula*), Stejneger's scoter (*Melanitta stejnegeri*), mallard (*Anas platyrhynchos*), ruddy shelduck (*Tadorna ferruginea*), common shelduck (*Tadorna tadorna*), common teal (*Anas crecca*), northern pintail (*Anas acuta*), northern shoveler (*Anas clypeata*), Eurasian wigeon (*Anas penelope*), eastern spot‐billed duck (*Anas poecilorhyncha*), common goldeneye (*Bucephala clangula*), tundra swan (*Cygnus columbianus*), whooper swan (*Cygnus cygnus*), red‐crested pochard (*Netta rufina*) and unidentified (*Anas spp and Anser spp*)] hosted 96.6% of all isolated viruses. Another 3.4% of isolates were obtained from Charadriiformes [spotted redshank (*Tringa erythropus*) and unidentified *Larus spp*.] and unknown species (Supporting Information S1).

## 4 DISCUSSION

It is generally accepted that wild birds represent the principal natural host of LPAIVs (Webster et al., 1992), with isolations documented from at least 105 wild bird species from 26 different taxonomic families (Olsen et al., 2006). In this study, the proportion of samples positive for AIV was relatively low (1.7%) and consistent across the different sampling periods and methodologies. This low proportion is consistent with previous studies that have been conducted in wild birds in Mongolia (Kang et al., 2011; Spackman et al., 2009). In our surveillance of Anseriformes and Charadriiformes orders, an absence of AIV‐linked wild bird mortality suggests detected viruses were LPAI. Many Anseriformes and Charadriiformes are known to make regular long‐distance migrations (Del Hoyo et al., 1992; Newton, 2008), and migratory birds play important roles in the geographic spread of AIV (Gilbert et al., 2012; Kang et al., 2011; Olsen et al., 2006; Reed et al., 2003). Numerous surveillance studies of wild ducks in the northern hemisphere have revealed higher LPAIV prevalence in juvenile birds with a peak in early autumn, when the birds start migrating south (Hinshaw et al., 1980). In North America, the prevalence falls from more than 60% in early autumn in ducks sampled at marshalling sites close to the Canadian breeding areas to 0.4% to 2% at the wintering grounds in the southern United States, and ∼0.25% when the ducks return to the northern breeding grounds in spring (Webster et al., 1992). Similar patterns have been observed in Northern Europe, although LPAIV detection during the spring migration can be significantly higher (Munster et al., 2007; Wallensten et al., 2006, 2007). Surveillance of the nesting grounds of ducks in Alaska and Siberia before winter migration revealed the presence of LPAIVs in 2.5% to 8% of birds (Okazaki et al., 2000). Differences in detection prevalence between the two time periods in May and August (Figure 2) appear to be affected by outbreaks with multiple positive samples for a single location (Figure 3). However, this does not explain the unusual lack of detections in the eastern region in October 2016–2018 compared with 2009–2013, when there were 17 unique subtype‐location events. October is historically the time when LPAIV events and prevalence are peaking (Munster et al., 2007; van Dijk et al., 2014; Wallensten et al., 2007). Additional research is needed but climatic variables such as temperature and precipitation have been described as important drivers of AIV in wild birds (Brown et al., 2009; Ferenczi et al., 2016; Pérez‐Ramírez et al., 2010; Si et al., 2010). The LPAIV variation between the time periods in October may be explained by the climatic conditions in the lakes. During this study, we were not able to estimate the ages of sampling target species. However, the proportion of AIV in autumn surveillance (2.5%) was significantly higher than in spring surveillance (0.6%). This result may be explained by the presence of large numbers of immunologically naïve juvenile birds from late summer onwards, which are more likely to be infected with LPAIVs.

Globally, a total of 112 AIV subtypes have been identified in wild birds, of which 49 have also been found in domestic species (Olson et al., 2014). Multiple AIV subtypes have been detected from wild birds in East Asia (Lee et al., 2017; Olson et al., 2014; Tang et al., 2020). In our study, 11 HA subtypes and 9 NA subtypes in 29 different combinations were detected in wild birds. The H1–H8, H10–H13 and H16 subtypes had already been isolated from wild birds in Mongolia (Hiono et al., 2015; Kang et al., 2011; Sharshov et al., 2014; Spackman et al., 2009; Ulaankhuu et al., 2020; Marchenko et al., 2010), but notably H13 and H16 were not isolated in this study. Among the subtypes, the most frequently isolated were H3N8 (39.4%), H4N6 (17.1%) and H10N7 (5.1%); these results are similar to the results reported from influenza virus surveillance conducted in Japan, South Korea, North America and European studies (Bui et al., 2011; Kang et al., 2011; Krauss et al., 2004; Munster et al., 2007; Otsuki et al., 1987). This finding indicates that AIVs have become mixed among different migratory bird species in overlapping flyways, driving the spread of the virus over long distances within Asia and between continents (Wang et al., 2008). Dabbling ducks of the *Anas* genus have been found to be infected with influenza viruses more frequently than other birds, including diving ducks (Olsen et al., 2006). We found AIVs in diving species and waders during 2009–2013 with species identification confirmed using DNA barcoding. H1N1, H2N5, H2N6, H4N6, H7N1 and H10N8 subtypes were found in diving ducks and waders, and all of these subtypes were also found in dabbling ducks except H2N6, which was detected only in a sample from a spotted redshank (Supporting Information S1). We isolated a single LPAI H5N1 virus subtype at Erdene Nuur (Lake) in October 2013, which is the first time this rare subtype has been isolated from wild birds in Mongolia. No clinical disease signs were observed in wild birds at the lake during sample collection, and the H5N1 virus had HA proteolytic cleavage sites consistent with LPAI viruses during the laboratory investigation. Although the majority of reported H5N1 are the HPAI type, a few cases of LPAI H5N1 in wild birds have been reported in Korea, China and North America (Duan et al., 2007; Kim et al., 2011; Spackman et al., 2007).

Mongolia is an important location in Asia for both wild bird habitat and migration, where our eight‐year study identified 175 LPAIVs of 29 subtypes from 10,222 samples and revealed geographic, taxonomic and seasonal patterns, as well as inter‐annual variation in prevalence. While no highly pathogenic AIVs were found during this period, detection in wild birds can help address threats to domestic animals and human health. This effort demonstrates the feasibility of surveillance and contributes to understanding the prevalence and ecology of LPAIVs in wild birds. Continued seasonal surveillance will provide additional information on the prevalence and virus circulation in the region. To understand global patterns of LPAI viruses in wild birds, it will be crucial to integrate investigations of viral and host ecology with long‐term surveillance studies, like this one, to provide more insight into the year‐round perpetuation of influenza viruses in wild birds (Olsen et al., 2006). In addition, long‐term surveillance efforts are important for providing ecological data on infections and for modelling disease spread for more precise risk analyses (Bevins et al., 2014).

## AUTHOR CONTRIBUTIONS

MG, LJ, AEF, JAKM, CKJ, ES and SHO developed the surveillance and sampling design. AB, MG, LJ, UA, DP, JMT, MS, BK and TS conducted the fieldwork to collect the fecal samples. JSMP, CYHL and UA performed the laboratory assays to identify AIVs, determine subtypes and analyse their pathogenicity. AB, MG, AEF, BD, BB, CYHL and SHO analysed and interpreted the fecal wild bird surveillance data. AB drafted the manuscript. MG, AEF, JAKM, CYHL and SHO reviewed and edited the final version of the manuscript.

## CONFLICT OF INTEREST STATEMENT

The authors declare no conflicts of interest.

### ETHICS STATEMENT

The non‐invasive environmental fecal sampling methodology did not require Institutional Animal Care and Use Committee (IACUC) approval. In 2016–2018, this non‐invasive methodology for wild birds was listed within a comprehensive animal sampling protocol reviewed and approved by the IACUC at the University of California at Davis (protocol number 16048).

### PEER REVIEW

The peer review history for this article is available at https://publons.com/publon/10.1002/vms3.1281.

## Supporting information

Supporting Information

## Data Availability

All data generated or analysed during this study are included in this published article (and its supporting information files). Additionally, 2016–2018 surveillance datasets generated and analysed during the current study are available in the USAID Data Development Library (DDL) repository, https://data.usaid.gov/d/tqea‐hwmr (PREDICT Consortium, 2021).
