# Supplementary material for: Ecological characterization of 175 low‐pathogenicity avian influenza viruses isolated from wild birds in Mongolia, 2009–2013 and 2016–2018
Source: Vet Med Sci. 2023 Sep 28;9(6):2676–85. doi: 10.1002/vms3.1281 (PMC10650234; doi:10.1002/vms3.1281)
Supplement: Supplementary file 1 — Supporting Information [file VMS3-9-2676-s001.docx]

**Appendices**

Supporting information 1. Detected LPAIVs from wild birds in Mongolia, 2009-2018

| **№** | **Species scientific name** | **Date** | **Subtype** | **Site name** | **Lat** | **Long** | **Region** |
| --- | --- | --- | --- | --- | --- | --- | --- |
| 1 | *Anas penelope* | 8/14/2009 | H4N6 | Khar Us Nuur | 47.939 | 91.988 | Western |
| 2 | *Netta rufina* | 8/17/2009 | H3N1 | Airag Nuur | 48.857 | 93.344 | Western |
| 3 | *Melanitta stejnegeri* | 9/15/2009 | H10N8 | Doroo Tsagaan Nuur | 49.058 | 101.163 | Central |
| 4 | *Melanitta stejnegeri* | 9/15/2009 | H10N8 | Doroo Tsagaan Nuur | 49.058 | 101.163 | Central |
| 5 | *Tadorna ferruginea* | 9/15/2009 | H3N8 | Doroo Tsagaan Nuur | 49.058 | 101.163 | Central |
| 6 | *Tadorna ferruginea* | 9/15/2009 | H3N8 | Doroo Tsagaan Nuur | 49.058 | 101.163 | Central |
| 7 | *Melanitta stejnegeri* | 9/15/2009 | H4N6 | Doroo Tsagaan Nuur | 49.058 | 101.163 | Central |
| 8 | *Tadorna ferruginea* | 9/15/2009 | H4N3 | Doroo Tsagaan Nuur | 49.058 | 101.163 | Central |
| 9 | *Anas clypeata* | 9/15/2009 | H3N6 | Doroo Tsagaan Nuur | 49.058 | 101.163 | Central |
| 10 | *Anas clypeata* | 9/15/2009 | H10N8 | Doroo Tsagaan Nuur | 49.058 | 101.163 | Central |
| 11 | *Unidentified duck spp.* | 9/15/2009 | H4N6 | Doroo Tsagaan Nuur | 49.058 | 101.163 | Central |
| 12 | *Anas clypeata* | 9/15/2009 | H3N8 | Doroo Tsagaan Nuur | 49.058 | 101.163 | Central |
| 13 | *Tadorna ferruginea* | 9/15/2009 | H3N8 | Doroo Tsagaan Nuur | 49.058 | 101.163 | Central |
| 14 | *Tadorna ferruginea* | 9/15/2009 | H3N8 | Doroo Tsagaan Nuur | 49.058 | 101.163 | Central |
| 15 | *Tadorna ferruginea* | 9/15/2009 | H4N6 | Doroo Tsagaan Nuur | 49.058 | 101.163 | Central |
| 16 | *Anas clypeata* | 9/15/2009 | H3N8 | Doroo Tsagaan Nuur | 49.058 | 101.163 | Central |
| 17 | *Anas clypeata* | 9/15/2009 | H3N8 | Doroo Tsagaan Nuur | 49.058 | 101.163 | Central |
| 18 | *Anas clypeata* | 9/15/2009 | H3N8 | Doroo Tsagaan Nuur | 49.058 | 101.163 | Central |
| 19 | *Unidentified duck spp.* | 9/15/2009 | H7N3 | Doroo Tsagaan Nuur | 49.058 | 101.163 | Central |
| 20 | *Unidentified duck spp.* | 9/15/2009 | H3N8 | Doroo Tsagaan Nuur | 49.058 | 101.163 | Central |
| 21 | *Anas platyrhynchos* | 6/19/2010 | H7N1 | Kherlen Gol | 48.608 | 115.410 | Eastern |
| 22 | *Tadorna ferruginea* | 6/22/2010 | H8N6 | Khaichiin Tsagaan Nuur | 49.676 | 114.658 | Eastern |
| 23 | *Tadorna ferruginea* | 6/22/2010 | H8N4 | Khaichiin Tsagaan Nuur | 49.676 | 114.658 | Eastern |
| 24 | *Anas platyrhynchos* | 6/22/2010 | H2N3 | Tergen Ikh Nuur | 49.397 | 113.241 | Eastern |
| 25 | *Tadorna ferruginea* | 7/10/2010 | H10N7 | Chuhiin Nuur | 49.532 | 114.676 | Eastern |
| 26 | *Tadorna ferruginea* | 7/10/2010 | H7N7 | Chuhiin Nuur | 49.532 | 114.676 | Eastern |
| 27 | *Tadorna ferruginea* | 7/24/2010 | H7N1 | Doroo Tsagaan Nuur | 49.058 | 101.163 | Central |
| 28 | *Tadorna ferruginea* | 7/24/2010 | H7N1 | Doroo Tsagaan Nuur | 49.058 | 101.163 | Central |
| 29 | *Bucephala clangula* | 7/24/2010 | H1N1 | Doroo Tsagaan Nuur | 49.058 | 101.163 | Central |
| 30 | *Bucephala clangula* | 7/24/2010 | H1N1 | Doroo Tsagaan Nuur | 49.058 | 101.163 | Central |
| 31 | *Bucephala clangula* | 7/24/2010 | H7N1 | Doroo Tsagaan Nuur | 49.058 | 101.163 | Central |
| 32 | *Anas platyrhynchos* | 9/10/2010 | H3N1 | Duut Nuur | 45.305 | 113.811 | Eastern |
| 33 | *Anas platyrhynchos* | 9/10/2010 | H3N1 | Duut Nuur | 45.305 | 113.811 | Eastern |
| 34 | *Anas platyrhynchos* | 9/10/2010 | H1N1 | Duut Nuur | 45.305 | 113.811 | Eastern |
| 35 | *Anas platyrhynchos* | 9/10/2010 | H3N8 | Duut Nuur | 45.305 | 113.811 | Eastern |
| 36 | *Tadorna tadorna* | 9/10/2010 | H1N1 | Ganga Nuur | 45.269 | 113.992 | Eastern |
| 37 | *Tadorna ferruginea* | 9/10/2010 | H4N6 | Ganga Nuur | 45.269 | 113.992 | Eastern |
| 38 | *Unidentified duck spp.* | 9/10/2010 | H4N6 | Ganga Nuur | 45.269 | 113.992 | Eastern |
| 39 | *Tadorna ferruginea* | 9/11/2010 | H10N8 | Erdene Nuur | 45.205 | 113.972 | Eastern |
| 40 | *Tadorna ferruginea* | 9/11/2010 | H10N8 | Erdene Nuur | 45.205 | 113.972 | Eastern |
| 41 | *Tadorna ferruginea* | 9/11/2010 | H3N1 | Erdene Nuur | 45.205 | 113.972 | Eastern |
| 42 | *Tadorna ferruginea* | 9/11/2010 | H4N6 | Erdene Nuur | 45.205 | 113.972 | Eastern |
| 43 | *Aythya fuligula* | 9/12/2010 | H1N1 | Doroo Tsagaan Nuur | 49.058 | 101.163 | Central |
| 44 | *Anas platyrhynchos* | 8/14/2011 | H4N6 | Davsan Tsagaan Nuur | 49.386 | 114.411 | Eastern |
| 45 | *Unidentified duck spp.* | 9/17/2011 | H3N2 | Erdene Nuur | 45.205 | 113.972 | Eastern |
| 46 | *Tadorna ferruginea* | 9/17/2011 | H3N8 | Erdene Nuur | 45.205 | 113.972 | Eastern |
| 47 | *Tadorna tadorna* | 9/17/2011 | H3N2 | Erdene Nuur | 45.205 | 113.972 | Eastern |
| 48 | *Anas crecca* | 9/17/2011 | H3N8 | Erdene Nuur | 45.205 | 113.972 | Eastern |
| 49 | *Anas crecca* | 9/17/2011 | H3N8 | Erdene Nuur | 45.205 | 113.972 | Eastern |
| 50 | *Anas crecca* | 9/17/2011 | H3N8 | Erdene Nuur | 45.205 | 113.972 | Eastern |
| 51 | *Anas crecca* | 9/18/2011 | H3N8 | Kholbo Nuur | 45.251 | 114.125 | Eastern |
| 52 | *Anas crecca* | 9/18/2011 | H3N8 | Kholbo Nuur | 45.251 | 114.125 | Eastern |
| 53 | *Anas crecca* | 9/18/2011 | H4N6 | Kholbo Nuur | 45.251 | 114.125 | Eastern |
| 54 | *Anas platyrhynchos* | 9/18/2011 | H3N8 | Kholbo Nuur | 45.251 | 114.125 | Eastern |
| 55 | *Tadorna tadorna* | 10/8/2011 | H4N6 | Ganga Nuur | 45.269 | 113.992 | Eastern |
| 56 | *Unidentified duck spp.* | 10/8/2011 | H5N3 | Ganga Nuur | 45.269 | 113.992 | Eastern |
| 57 | *Tadorna tadorna* | 10/8/2011 | H3N8 | Ganga Nuur | 45.269 | 113.992 | Eastern |
| 58 | *Tadorna tadorna* | 10/9/2011 | H3N8 | Duut Nuur | 45.305 | 113.811 | Eastern |
| 59 | *Tadorna tadorna* | 10/9/2011 | H4N6 | Duut Nuur | 45.305 | 113.811 | Eastern |
| 60 | *Tadorna tadorna* | 10/9/2011 | H4N6 | Duut Nuur | 45.305 | 113.811 | Eastern |
| 61 | *Tadorna tadorna* | 10/9/2011 | H3N3 | Duut Nuur | 45.305 | 113.811 | Eastern |
| 62 | *Tadorna tadorna* | 10/9/2011 | H3N8 | Duut Nuur | 45.305 | 113.811 | Eastern |
| 63 | *Tadorna tadorna* | 10/9/2011 | H3N8 | Duut Nuur | 45.305 | 113.811 | Eastern |
| 64 | *Tadorna tadorna* | 10/9/2011 | H5N3 | Duut Nuur | 45.305 | 113.811 | Eastern |
| 65 | *Anas crecca* | 10/9/2011 | H3N8 | Duut Nuur | 45.305 | 113.811 | Eastern |
| 66 | *Anas crecca* | 10/10/2011 | H3N8 | Kholbo Nuur | 45.251 | 114.125 | Eastern |
| 67 | *Anas crecca* | 10/10/2011 | H6N6 | Kholbo Nuur | 45.251 | 114.125 | Eastern |
| 68 | *Anas crecca* | 10/10/2011 | H4N6 | Kholbo Nuur | 45.251 | 114.125 | Eastern |
| 69 | *Anas platyrhynchos* | 10/11/2011 | H4N6 | Erdene Nuur | 45.205 | 113.972 | Eastern |
| 70 | *Anas platyrhynchos* | 9/5/2012 | H11N9 | Turgenii Ikh Nuur | 49.396 | 113.260 | Eastern |
| 71 | *Anas platyrhynchos* | 9/5/2012 | H3N8 | Turgenii Ikh Nuur | 49.396 | 113.260 | Eastern |
| 72 | *Anas platyrhynchos* | 9/7/2012 | H1N1 | Ganga Nuur | 45.269 | 113.992 | Eastern |
| 73 | *Tadorna ferruginea* | 9/7/2012 | H3N9 | Ganga Nuur | 45.269 | 113.992 | Eastern |
| 74 | *Tadorna ferruginea* | 9/7/2012 | H3N8 | Ganga Nuur | 45.269 | 113.992 | Eastern |
| 75 | *Anas clypeata* | 9/7/2012 | H3N8 | Duut Nuur | 45.305 | 113.811 | Eastern |
| 76 | *Anas clypeata* | 9/7/2012 | H4N6 | Duut Nuur | 45.305 | 113.811 | Eastern |
| 77 | *Anas platyrhynchos* | 9/7/2012 | H4N6 | Duut Nuur | 45.305 | 113.811 | Eastern |
| 78 | *Anas platyrhynchos* | 9/7/2012 | H3N8 | Duut Nuur | 45.305 | 113.811 | Eastern |
| 79 | *Anas platyrhynchos* | 9/8/2012 | H3N8 | Kholbo Nuur | 45.251 | 114.125 | Eastern |
| 80 | *Unidentified duck spp* | 9/8/2012 | H3N1 | Kholbo Nuur | 45.251 | 114.125 | Eastern |
| 81 | *Anas platyrhynchos* | 10/3/2012 | H11N2 | Tsagaan Nuur | 45.338 | 113.316 | Eastern |
| 82 | *Anas platyrhynchos* | 10/4/2012 | H3N2 | Duut Nuur | 45.305 | 113.811 | Eastern |
| 83 | *Anas crecca* | 10/5/2012 | H6N2 | Ganga Nuur | 45.269 | 113.992 | Eastern |
| 84 | *Anas crecca* | 10/5/2012 | H3N8 | Ganga Nuur | 45.269 | 113.992 | Eastern |
| 85 | *Tringa erythropus* | 10/1/2013 | H2N6 | Duut Nuur | 45.305 | 113.811 | Eastern |
| 86 | *Tringa erythropus* | 10/1/2013 | H2N5 | Duut Nuur | 45.305 | 113.811 | Eastern |
| 87 | *Anas acuta* | 10/2/2013 | H4N6 | Ganga Nuur | 45.269 | 113.992 | Eastern |
| 88 | *Cygnus columbianus* | 10/2/2013 | H12N5 | Ganga Nuur | 45.269 | 113.992 | Eastern |
| 89 | *Tadorna tadorna* | 10/2/2013 | H1N1 | Ganga Nuur | 45.269 | 113.992 | Eastern |
| 90 | *Tadorna tadorna* | 10/2/2013 | H3N8 | Ganga Nuur | 45.269 | 113.992 | Eastern |
| 91 | *Tadorna tadorna* | 10/2/2013 | H4N6 | Ganga Nuur | 45.269 | 113.992 | Eastern |
| 92 | *Tadorna tadorna* | 10/2/2013 | H3N8 | Ganga Nuur | 45.269 | 113.992 | Eastern |
| 93 | *Anas platyrhynchos* | 10/4/2013 | H2N5 | Erdene Nuur | 45.205 | 113.972 | Eastern |
| 94 | *Tringa erythropus* | 10/4/2013 | H2N6 | Erdene Nuur | 45.205 | 113.972 | Eastern |
| 95 | *Tringa erythropus* | 10/4/2013 | H2N6 | Erdene Nuur | 45.205 | 113.972 | Eastern |
| 96 | *Unidentified duck spp.* | 10/4/2013 | H2N5 | Erdene Nuur | 45.205 | 113.972 | Eastern |
| 97 | *Anas poecilorhyncha* | 10/4/2013 | H2N5 | Erdene Nuur | 45.205 | 113.972 | Eastern |
| 98 | *Anas platyrhynchos* | 10/4/2013 | H2N5 | Erdene Nuur | 45.205 | 113.972 | Eastern |
| 99 | *Anas platyrhynchos* | 10/4/2013 | H2N5 | Erdene Nuur | 45.205 | 113.972 | Eastern |
| 100 | *Tadorna ferruginea* | 10/19/2013 | H3N2 | Duut Nuur | 45.305 | 113.811 | Eastern |
| 101 | *Unidentified duck spp.* | 10/19/2013 | H3N8 | Ganga Nuur | 45.269 | 113.992 | Eastern |
| 102 | *Tadorna tadorna* | 10/19/2013 | H1N1 | Ganga Nuur | 45.269 | 113.992 | Eastern |
| 103 | *Tadorna ferruginea* | 10/20/2013 | H3N5 | Kholbo Nuur | 45.251 | 114.125 | Eastern |
| 104 | *Tadorna ferruginea* | 10/20/2013 | H3N5 | Kholbo Nuur | 45.251 | 114.125 | Eastern |
| 105 | *Tadorna ferruginea* | 10/20/2013 | H3N8 | Kholbo Nuur | 45.251 | 114.125 | Eastern |
| 106 | *Tadorna ferruginea* | 10/20/2013 | H3N8 | Kholbo Nuur | 45.251 | 114.125 | Eastern |
| 107 | *Tadorna ferruginea* | 10/20/2013 | H12N1 | Kholbo Nuur | 45.251 | 114.125 | Eastern |
| 108 | *Tadorna ferruginea* | 10/20/2013 | H3N8 | Kholbo Nuur | 45.251 | 114.125 | Eastern |
| 109 | *Tadorna ferruginea* | 10/20/2013 | H3N8 | Kholbo Nuur | 45.251 | 114.125 | Eastern |
| 110 | *Unidentified duck spp.* | 10/20/2013 | H12N1 | Kholbo Nuur | 45.251 | 114.125 | Eastern |
| 111 | *Tadorna ferruginea* | 10/20/2013 | H2N2 | Kholbo Nuur | 45.251 | 114.125 | Eastern |
| 112 | *Tadorna ferruginea* | 10/20/2013 | H3N5 | Kholbo Nuur | 45.251 | 114.125 | Eastern |
| 113 | *Tadorna ferruginea* | 10/20/2013 | H3N6 | Erdene Nuur | 45.205 | 113.972 | Eastern |
| 114 | *Tadorna ferruginea* | 10/20/2013 | H3N8 | Erdene Nuur | 45.205 | 113.972 | Eastern |
| 115 | *Anas platyrhynchos* | 10/20/2013 | H2N5 | Erdene Nuur | 45.205 | 113.972 | Eastern |
| 116 | *Anas poecilorhyncha* | 10/20/2013 | H3N2 | Erdene Nuur | 45.205 | 113.972 | Eastern |
| 117 | *Cygnus cygnus* | 10/20/2013 | H5N1 | Erdene Nuur | 45.205 | 113.972 | Eastern |
| 118 | *Tadorna ferruginea* | 10/21/2013 | H3N8 | Duut Nuur | 45.305 | 113.811 | Eastern |
| 119 | *Cygnus spp.* | 5/26/2016 | H3N8 | Doitiin Tsagaan Nuur | 47.569 | 102.524 | Central |
| 120 | *Cygnus spp.* | 5/26/2016 | H3N8 | Doitiin Tsagaan Nuur | 47.569 | 102.524 | Central |
| 121 | *Cygnus spp.* | 5/26/2016 | H3N8 | Doitiin Tsagaan Nuur | 47.569 | 102.524 | Central |
| 122 | *Cygnus spp.* | 5/26/2016 | H3N8 | Doitiin Tsagaan Nuur | 47.569 | 102.524 | Central |
| 123 | *Cygnus spp.* | 5/26/2016 | H3N8 | Doitiin Tsagaan Nuur | 47.569 | 102.524 | Central |
| 124 | *Cygnus spp.* | 5/26/2016 | H3N8 | Doitiin Tsagaan Nuur | 47.569 | 102.524 | Central |
| 125 | *Anas spp.* | 5/26/2016 | H3N8 | Ugii Nuur | 47.770 | 102.770 | Central |
| 126 | *Anas spp.* | 5/26/2016 | H3N8 | Ugii Nuur | 47.770 | 102.770 | Central |
| 127 | *Anas spp.* | 5/26/2016 | H3N8 | Ugii Nuur | 47.770 | 102.770 | Central |
| 128 | *Anas spp.* | 5/26/2016 | H3N8 | Ugii Nuur | 47.770 | 102.770 | Central |
| 129 | *Anas spp.* | 5/26/2016 | H3N8 | Ugii Nuur | 47.770 | 102.770 | Central |
| 130 | *Anas spp.* | 5/26/2016 | H3N8 | Ugii Nuur | 47.770 | 102.770 | Central |
| 131 | *Anser spp.* | 5/31/2016 | H6N2 | Khar Us Nuur | 47.851 | 92.022 | Western |
| 132 | *Anser spp.* | 5/31/2016 | H6N2 | Khar Us Nuur | 47.851 | 92.022 | Western |
| 133 | *Anser spp.* | 5/31/2016 | H6N2 | Khar Us Nuur | 47.851 | 92.022 | Western |
| 134 | *Tadorna spp.* | 9/8/2016 | H3N8 | Doitiin Tsagaan Nuur | 47.569 | 102.524 | Central |
| 135 | *Tadorna spp.* | 9/8/2016 | H3N8 | Doitiin Tsagaan Nuur | 47.569 | 102.524 | Central |
| 136 | *Tadorna spp.* | 9/8/2016 | H3N8 | Doitiin Tsagaan Nuur | 47.569 | 102.524 | Central |
| 137 | *Anas spp.* | 9/10/2016 | H10N7 | Terkhiin Tsagaan Nuur | 48.131 | 99.636 | Central |
| 138 | *Tadorna spp.* | 9/12/2016 | H10N7 | Small Nuur | 48.589 | 97.515 | Central |
| 139 | *Tadorna spp.* | 9/12/2016 | H10N7 | Small Nuur | 48.589 | 97.515 | Central |
| 140 | *Tadorna spp.* | 9/12/2016 | H10N7 | Small Nuur | 48.589 | 97.515 | Central |
| 141 | *Tadorna spp.* | 9/12/2016 | H10N7 | Small Nuur | 48.589 | 97.515 | Central |
| 142 | *Tadorna spp.* | 9/12/2016 | H10N7 | Small Nuur | 48.589 | 97.515 | Central |
| 143 | *Tadorna spp.* | 9/12/2016 | H10N7 | Small Nuur | 48.589 | 97.515 | Central |
| 144 | *Tadorna spp.* | 9/12/2016 | H10N7 | Small Nuur | 48.589 | 97.515 | Central |
| 145 | *Tadorna spp.* | 9/20/2016 | H3N8 | Erkhel Nuur | 49.933 | 99.928 | Central |
| 146 | *Tadorna spp.* | 9/20/2016 | H3N8 | Erkhel Nuur | 49.933 | 99.928 | Central |
| 147 | *Tadorna spp.* | 9/23/2016 | H4N6 | Doroo Tsagaan Nuur | 49.024 | 101.218 | Central |
| 148 | *Tadorna spp.* | 9/23/2016 | H4N6 | Doroo Tsagaan Nuur | 49.024 | 101.218 | Central |
| 149 | *Tadorna spp.* | 9/23/2016 | H4N6 | Doroo Tsagaan Nuur | 49.024 | 101.218 | Central |
| 150 | *Tadorna spp.* | 9/23/2016 | H4N6 | Doroo Tsagaan Nuur | 49.024 | 101.218 | Central |
| 151 | *Tadorna spp.* | 9/23/2016 | H4N6 | Doroo Tsagaan Nuur | 49.024 | 101.218 | Central |
| 152 | *Tadorna spp.* | 9/23/2016 | H4N6 | Doroo Tsagaan Nuur | 49.024 | 101.218 | Central |
| 153 | *Tadorna spp.* | 9/23/2016 | H4N6 | Doroo Tsagaan Nuur | 49.024 | 101.218 | Central |
| 154 | *Tadorna spp.* | 9/23/2016 | H4N6 | Doroo Tsagaan Nuur | 49.024 | 101.218 | Central |
| 155 | *Tadorna spp.* | 9/24/2016 | H3N8 | Sharga Nuur | 48.946 | 101.952 | Central |
| 156 | *Tadorna spp.* | 9/24/2016 | H3N8 | Sharga Nuur | 48.946 | 101.952 | Central |
| 157 | *Tadorna spp.* | 9/24/2016 | H3N8 | Sharga Nuur | 48.946 | 101.952 | Central |
| 158 | *Anas spp.* | 9/25/2016 | H3N8 | Khunt Nuur | 48.433 | 102.580 | Central |
| 159 | *Anas spp.* | 9/25/2016 | H3N8 | Khunt Nuur | 48.433 | 102.580 | Central |
| 160 | *Anas spp.* | 9/25/2016 | H3N8 | Khunt Nuur | 48.433 | 102.580 | Central |
| 161 | *Anas spp.* | 9/25/2016 | H3N8 | Khunt Nuur | 48.433 | 102.580 | Central |
| 162 | *Anas spp.* | 9/25/2016 | H3N8 | Khunt Nuur | 48.433 | 102.580 | Central |
| 163 | *Anas spp.* | 9/25/2016 | H3N8 | Khunt Nuur | 48.433 | 102.580 | Central |
| 164 | *Anas spp.* | 9/25/2016 | H3N8 | Khunt Nuur | 48.433 | 102.580 | Central |
| 165 | *Tadorna spp.* | 5/1/2017 | H2N2 | Ganga Nuur | 45.258 | 113.991 | Eastern |
| 166 | *Larus spp.* | 6/11/2017 | H2N2 | Tes River Delta | 50.462 | 93.126 | Western |
| 167 | *Anas spp.* | 8/30/2017 | H4N6 | Zost Nuur | 48.881 | 93.303 | Western |
| 168 | *Anas spp.* | 8/30/2017 | H4N6 | Zost Nuur | 48.881 | 93.303 | Western |
| 169 | *Anas spp.* | 8/30/2017 | H4N6 | Zost Nuur | 48.881 | 93.303 | Western |
| 170 | *Anas spp.* | 8/30/2017 | H4N6 | Zost Nuur | 48.881 | 93.303 | Western |
| 171 | *Anas spp.* | 6/10/2018 | H4N6 | Bayan Nuur | 47.855 | 104.308 | Central |
| 172 | *Anas spp.* | 8/22/2018 | H3N8 | Doitiin Tsagaan Nuur | 47.569 | 102.524 | Central |
| 173 | *Anas spp.* | 8/22/2018 | H3N8 | Doitiin Tsagaan Nuur | 47.569 | 102.524 | Central |
| 174 | *Anas spp.* | 8/22/2018 | H3N8 | Doitiin Tsagaan Nuur | 47.569 | 102.524 | Central |
| 175 | *Anas spp.* | 8/22/2018 | H3N8 | Doitiin Tsagaan Nuur | 47.569 | 102.524 | Central |
